# Isolation and Characterization of Highly Replicable Hepatitis C Virus Genotype 1a Strain HCV-RMT

**DOI:** 10.1371/journal.pone.0082527

**Published:** 2013-12-16

**Authors:** Masaaki Arai, Yuko Tokunaga, Asako Takagi, Yoshimi Tobita, Yuichi Hirata, Yuji Ishida, Chise Tateno, Michinori Kohara

**Affiliations:** 1 Advanced Medical Research Laboratory, Mitsubishi Tanabe Pharma Corporation, Kanagawa, Japan; 2 Department of Microbiology and Cell Biology, Tokyo Metropolitan Institute of Medical Science, Tokyo, Japan; 3 PhoenixBio Co., Ltd., Hiroshima, Japan; University of Washington, United States of America

## Abstract

Multiple genotype 1a clones have been reported, including the very first hepatitis C virus (HCV) clone called H77. The replication ability of some of these clones has been confirmed *in vitro* and *in vivo*, although this ability is somehow compromised. We now report a newly isolated genotype 1a clone, designated HCV-RMT, which has the ability to replicate efficiently in patients, chimeric mice with humanized liver, and cultured cells. An authentic subgenomic replicon cell line was established from the HCV-RMT sequence with spontaneous introduction of three adaptive mutations, which were later confirmed to be responsible for efficient replication in HuH-7 cells as both subgenomic replicon RNA and viral genome RNA. Following transfection, the HCV-RMT RNA genome with three adaptive mutations was maintained for more than 2 months in HuH-7 cells. One clone selected from the transfected cells had a high copy number, and its supernatant could infect naïve HuH-7 cells. Direct injection of wild-type HCV-RMT RNA into the liver of chimeric mice with humanized liver resulted in vigorous replication, similar to inoculation with the parental patient’s serum. A study of virus replication using HCV-RMT derivatives with various combinations of adaptive mutations revealed a clear inversely proportional relationship between *in vitro* and *in vivo* replication abilities. Thus, we suggest that HCV-RMT and its derivatives are important tools for HCV genotype 1a research and for determining the mechanism of HCV replication *in vitro* and *in vivo*.

## Introduction

Hepatitis C virus (HCV) is an enveloped positive-strand RNA virus that belongs to the Flaviviridae family [1]. HCV infection is a major cause of chronic hepatitis, liver cirrhosis, and hepatocellular carcinoma. With over 170 million people currently infected worldwide [2], HCV represents a growing public health burden despite the launch of new antiviral medications that directly inhibit virus replication [3], [4].

Since HCV was first identified in 1989 as the major cause of non-A and non-B hepatitis [5], great progress has been made in understanding the life cycle of HCV. The first propagation system for this disease agent was an *in vivo* chimpanzee model [6], [7], [8]. Although that system is still occasionally used as a pivotal animal model for some drugs, chimeric mice with humanized liver that is generated by transplanting human hepatocytes [9], [10] are more popular now because of the low cost and the absence of ethical concerns associated with the use of chimpanzees. For *in vitro* research, establishment of an HCV replicon system [11], [12] was an important achievement that allowed research into the function of individual non-structural viral proteins. However, the entire viral life cycle remains enigmatic because no structural proteins are needed in this system. Some reports have been published about full-length replicons with structural proteins in addition to non-structural proteins, although little [13] or no [14], [15] secretion of infectious virions was observed, which may have been partly due to adaptive mutations. Another breakthrough was made with the discovery of a genotype 2a Japan fulminant hepatitis (JFH)-1 strain that soon became well known for its vigorous replication as a replicon with no adaptive mutations [16]. JFH-1 can also infect and propagate in cultured cells as a virus, especially in HuH-7 cells or their derivatives [17]–[19]. After the discovery of JFH-1, two methods were available for the investigation of how viral proteins other than those of HCV genotype 2a function during their entire life cycle. The first method was only for structural proteins and involved making a hybrid of the structural region of the clone of interest and the non-structural regions of JFH-1 for efficient replication [20]–[22]. The other method utilized the entire viral genome sequence of genotype 1 and made them infectious to HuH-7 derivative cells by introducing known adaptive mutations [23], [24] or enhancing replication with a casein kinase inhibitor [25]; however, their replication abilities were somehow compromised. In this study, we report the isolation of a new genotype 1a strain from a patient’s serum sample that was highly infectious to human hepatocyte-transplanted chimeric mice, as the viral titer in the blood of the mice was higher than 10^8^ copies/ml. We evaluated its replication abilities in four replication systems: subgenomic replicon, virus, *in vitro* infection, and *in vivo* infection. The new HCV clone, which was designated HCV-RMT (GenBank accession number, AB520610), was different from other genotype 1a clones because it did not require any artificially introduced adaptive mutations for the establishment of replicon cells. With these features, our newly cloned HCV-RMT may be a useful tool for investigating the entire life cycle of genotype 1 HCV.

## Materials and Methods

### Ethics Statement

This study was carried out in strict accordance with both the *Guidelines for Animal Experimentation* of the Japanese Association for Laboratory Animal Science and the recommendations in the *Guide for the Care and Use of Laboratory Animals* of the National Institutes of Health. All protocols were approved by the ethics committee of Tokyo Metropolitan Institute of Medical Science.

### Cloning and Sequencing

Acute-phase serum from an HCV genotype 1a-infected patient, HCG9 (purchased from International Reagents Corp., Kobe, Japan; discontinued), was supplemented with 0.1 µg/µl yeast tRNA, and total RNA was extracted using ISOGEN-LS (Nippon Gene, Tokyo, Japan) according to the manufacturer’s information. Purified RNA (1 µg) was reverse transcribed using LongRange Reverse transcriptase (QIAGEN, Valencia, CA, USA) and a 21-mer oligonucleotide (antisense sequence 9549-9569 of HCV-H77: GenBank accession number AF011751) as the primer. The first PCR amplification was carried out with the generated cDNA and Phusion DNA polymerase (Finnzymes, Vantaa, Finland) using sense primers corresponding to nucleotides 9-28, 2952-2972, and 5963-5979 (numbers correspond to the HCV-H77 sequence) and antisense primers corresponding to nucleotides 4038-4054, 7042-7057, and 9549-9569. The second nested PCR amplification was carried out with these three products using sense primers corresponding to nucleotides 23-43, 2967-2987, and 5981-6000 and antisense primers corresponding to nucleotides 4018-4033, 7016-7035, and 9534-9554. For the cloning of terminals, total RNA was purified from non-supplemented HCG9 serum. The 5′ terminus was amplified with a 5′ RACE system kit (Invitrogen, Carlsbad, CA, USA) using one-fourth of the purified total RNA from 100 µl serum and antisense primers corresponding to nucleotides 255–273 for the first PCR and 241–261 for the second nested PCR. For the 3′ terminus, the poly(A) tail was added to the 3′ terminus of the same amount of RNA with poly(A) polymerase (Takara Bio Inc., Shiga, Japan). Reverse transcription and PCR amplification of this region were carried out using oligo-d(T) as the reverse primer for both reactions and primers corresponding to nucleotides 9385-9408 for PCR.

All fragments were subcloned using a TOPO cloning kit (Invitrogen), and sequences yielding 10 or more clones per fragment were determined with the Big Dye Terminator mix and ABIprism3100 (Applied Biosystems, Foster City, CA, USA). The consensus sequence was determined by accepting the most frequent nucleotide at each position.

### Construction and RNA transcription

To generate full-length viral RNA, the HCV-RMT sequence, which has an endogenous *Xba*I site, was mutated to a silent mutation (T3941C) using a QuikChangeII kit (Stratagene, La Jolla, CA, USA) and cloned into the *Hind*III site of pBR322 with an additional T7 promoter at the beginning and an *Xba*I site at the end. Replicon construction of HCV-RMT was performed by replacing nucleotides 390-3419 of HCV-RMT with the neomycin resistance gene, encephalomyocarditis virus internal ribosome entry site (EMCV-IRES), and an additional start codon at the beginning of the NS3 region. For RNA generation, plasmids were digested with *Xba*I and used as a template for RNA transcription using a RiboMax kit (Promega, Madison, WI, USA).

### Cells and Electroporation

HuH-7 cells were cultured in DMEM-GlutaMax-I (Invitrogen) supplemented with 10% fetal bovine serum, penicillin, and streptomycin (Invitrogen). Replicon cells were maintained in the same medium supplemented with 300 µg/ml G418 (Invitrogen). Cells were passaged three times a week at a split of four times. Electroporation of replicon RNA and G418 selection were performed as previously described [11], [12]. The cured replicon cell clone (HuH7-K4) was established as previously described [13]. Briefly, authentic subgenomic replicon cells were treated with 1000 IU interferon-α (Mochida Pharmaceutical Co., Ltd., Tokyo, Japan) for 2 months and cloned using the limiting dilution method.

### Quantification of HCV RNA

Total RNA was purified from 1 µl chimeric mouse serum using SepaGene RV-R (Sanko Junyaku, Tokyo, Japan), and total RNA was prepared from cells or liver tissues using the acid guanidium thiocyanate-phenol-chloroform extraction method. Quantification of HCV RNA copy number with real-time RT-PCR was performed using an ABI 7700 system (Applied Biosystems) as described previously [26].

### Western blot analysis and immunofluorescence analysis

Western blot analysis was carried out according to the conventional semi-dry blot method. Cells were lysed with lysis buffer (10 mM Tris-HCl, pH 7.4 containing 1% sodium dodecyl sulfate, 0.5% Nonidet P-40, 150 mM NaCl, 0.5 mM EDTA, and 1 mM dithiothreitol). Protein (10 µg) from each sample was separated with SDS-PAGE through a 10% polyacrylamide gel and transferred to a polyvinylidene difluoride membrane, Immobilon-P (Millipore, Billerica, MA, USA). HCV NS3 protein was detected with 5 µg/ml anti-NS3 polyclonal antibody (R212) as described previously [27]. HCV NS5B protein and β-actin were detected with 0.5 µg/ml anti-NS5B polyclonal antibody (ab35586; Abcam, Cambridge, UK) and 0.2 µg/ml anti-β-actin monoclonal antibody (AC-15; Sigma-Aldrich, St. Louis, MO, USA), respectively.

For immunofluorescence analysis, cells were washed twice with PBS(−) and fixed with 100% methanol (chilled at −80°C) at −20°C for 20 min. Fixed cells were treated with PBS(−) supplemented with 1% BSA and 2.5 mM EDTA overnight at 4°C. Blocking and antibody treatments were also carried out in the same buffer. Stained cells were viewed with a laser scanning confocal microscope LSM510 (Carl Zeiss, Oberkochen, Germany). HCV core proteins were detected with 5 µg/ml α-HCV core monoclonal antibody (31-2) prepared in our laboratory [28].

### *In vitro* infection and α-CD81 blocking

HuH7-K4 cells were seeded at 6×10^4^ cells/well onto a *φ*10-mm coverglass in a 48-well plate 24 h before inoculation with 240 µl culture medium. At 72 h post-inoculation, cells were fixed with 100% methanol (chilled to −80°C) for 20 min at −20°C. HCV core proteins were detected with 5 µg/ml α-HCV core antibody 31-2. Fluorescent-positive foci were counted under fluorescence microscopy, and the focus-forming units (ffu) per milliliter of supernatant were calculated.

For α-CD81 blocking, HuH7-K4 cells were pre-treated with a serial dilution of α-CD81 antibody (JS81, BD Pharmingen, San Diego, CA, USA) or normal mouse IgG_1_ (BD Pharmingen) as an isotype control for 1 h before inoculation.

### Drug treatment

#11 cells (5,000 cells/well), which were established using the single cell cloning of HCV-RMTtri-electroporated cells, were seeded in 96-well tissue culture plates and cultivated overnight. Serial dilutions of cyclosporin A (Fluka Chemie, Buchs, Switzerland) or interferon-α (Mochida Pharmaceutical Co., Ltd.) were added. After incubation for 72 h, total RNA was extracted from cells, and HCV-RNA was quantified as described above. The experiments were carried out in triplicate.

### *In vivo* infection

Chimeric mice with humanized liver (PhoenixBio, Hiroshima, Japan) were infected with 10 µl patient serum HCG9 by intravenous injection. For analysis of infectivity of the HCV genome clone, mice were directly injected with 30 µg of the generated RNAs into five to six sites in the liver during abdominal surgery. Blood samples were collected once a week and used for quantification of HCV copy number.

## Results

### Cloning of a new HCV genotype 1a strain from the serum of an HCG9-infected mouse

**Figure 1:**
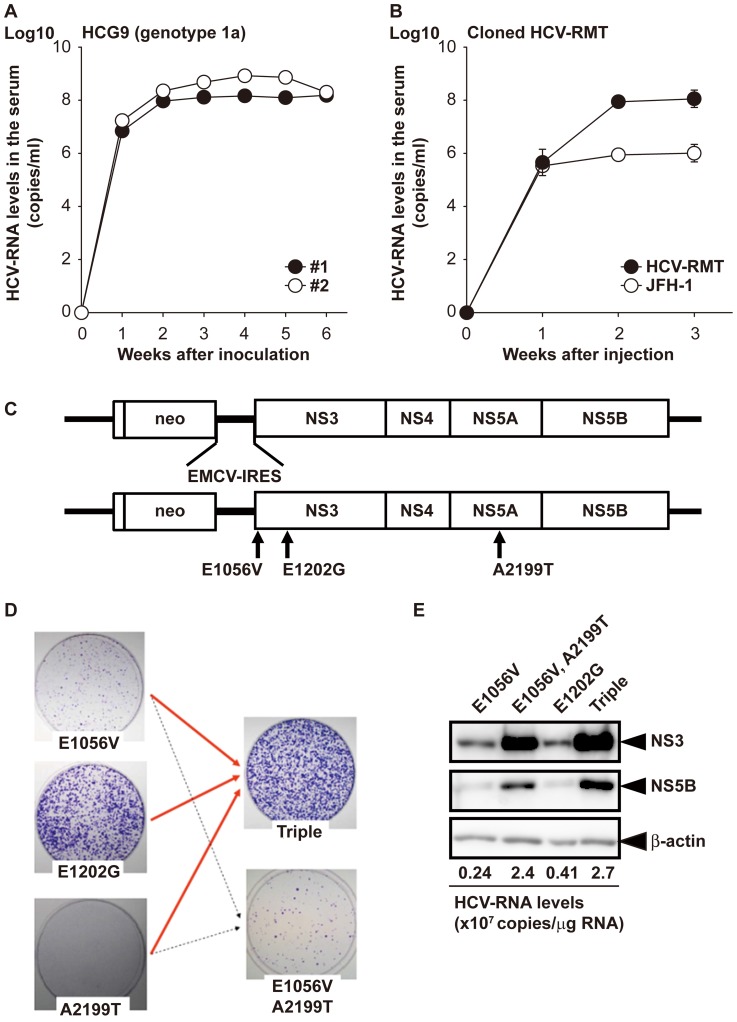
Basic characteristics of the HCV-RMT clone. Change in HCV copy number in chimeric mice. (A) Two mice were intravenously infected with 10 µl patient serum HCG9. (B) Three mice per group were directly injected with 30 µg HCV RNAs of the HCV-RMT strain or the JFH-1 strain into the liver. Data are indicated as the mean ± S.D. (C) Schematic representation of construction of the replicon and the sites of adaptive mutations. (D) Colony formation assay of replicon clones with adaptive mutations. Each RNA (1 µg) was electroporated into HuH7-K4 cells. (E) Western blot analysis of replicon cells. Each culture of replicon RNA-electroporated cells was maintained and passaged with G418 selection for 2 weeks. Cell lysates (10 µg) were loaded onto an SDS-PAGE gel.

We first infected chimeric mice with humanized liver with patient serum HCG9. HCV in HCG9 serum was classified as genotype 1a with RT-PCR genotyping and showed a relatively high replication ability in the patient and a comparable or better replication ability in the chimeric mice (Figure 1A). In one infected mouse, the HCV copy number in blood reached 1×10^9^/ml (data not shown). Using two mice with blood titers of 1×10^8^ and 1×10^9^ copies/ml, we cloned HCV sequences with the standard PCR amplification method using HCV-H77 as a source of primer sequences. Except for some length variations in the poly-pyrimidine tract region, we found no differences in HCV sequences from mouse blood with titers of 1×10^8^ and 1×10^9^ copies/ml when considering major consensus nucleotides at all sites (GenBank accession number AB520610). The HCV sequences were identical to the HCV sequence cloned from HCG9 serum itself (data not shown). We designated this sequence as HCV-RMT. Its homology to the HCV-H77 strain was 92.8% for nucleotides and 95.1% for amino acids. The *in vivo* replication ability was confirmed with direct injection of the generated HCV-RMT RNA genome into livers of the chimeric mice. Blood titers were comparable to infection with parental HCG9 serum (Figure 1B). JFH-1 infection resulted in a 2-log lower blood titer than HCV-RMT when the same procedure was used.

### Establishment of subgenomic replicon cells with the HCV-RMT strain

Next, we generated an authentic subgenomic replicon RNA construct using the HCV-RMT sequence and used it to establish replicon cells. Only two colonies appeared after electroporation with 30 µg of the HCV-RMT replicon RNA and G418 selection. One of these colonies had a reasonable HCV subgenome copy number, and thus, we propagated it and determined the sequence of the subgenome. The determined consensus sequence of the subgenome had three mutations from the wild type: two were located in the NS3 region (E1056V and E1202G), and one was in the NS5A region (A2199T) (Figure 1C). We introduced these mutations into the HCV-RMT replicon sequence as a single mutation or combination of mutations and identified the mutations that were responsible for colony formation (Figure 1D). The most influential single mutation was E1202G in the NS3 region, although a combination of all three mutations (designated RMTtri) resulted in the best replication ability. Interestingly, western blot analysis and HCV genome quantification revealed that the amount of HCV viral components in cells was independent of the colony-forming ability and seemed to be negatively affected by the most beneficial adaptive mutation (E1202G) (Figure 1E).

### The HCV-RMT RNA genome with adaptive mutations was maintained in HuH-7 cells

**Figure 2:**
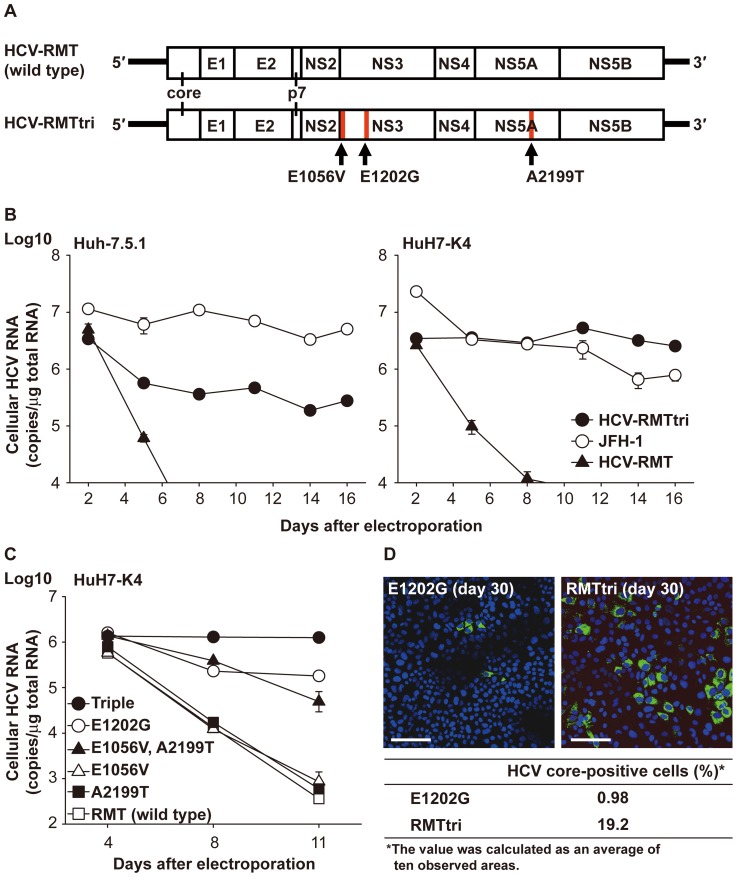
*In vitro* replication ability of HCV-RMT derivative genomes. (A) Schematic representation of construction of the HCV genome and the sites of adaptive mutations (red bars). (B) Electroporation of the generated HCV-RNA genomes of wild-type HCV-RMT (closed triangles), HCV-RMT with triple mutations (HCV-RMTtri; closed circles), and the JFH-1 strain (open circles) into Huh-7.5.1 or HuH7-K4 cells. The experiments were carried out in duplicate. (C) Comparison of the *in vitro* replication ability of each HCV-RMT derivative in HuH7-K4 cells. The experiments were carried out in duplicate. Wild type: open squares, E1202G: open circles, E1056V: open triangles, A2199T: closed squares, E1056V and A2199T: closed triangles, triple mutations: closed circles. (D) Immunostaining for the HCV core protein in HCV-RNA-electroporated cells. Scale bar  =  100 µm. The percent of HCV core protein-positive cells (%) was calculated as an average of ten observed areas.

Next, we assessed the *in vitro* replication abilities of HCV-RMT derivatives as a viral genome rather than a replicon. We introduced adaptive mutation(s) into the HCV-RMT sequence (Figure 2A) and electroporated the *in vitro*-generated RNAs into Huh-7.5.1 and HuH7-K4 cells. Electroporated cells were passaged every 2 to 4 days depending on their confluency, and sampling of cells for quantification of the HCV RNA genome was carried out at each passage. The amounts of HCV-RMTtri and JFH-1 were maintained at ≥1×10^5^ copies/µg total RNA, in contrast to wild-type HCV-RMT, which was eliminated rapidly (Figure 2B). Additionally, different cell preferences were observed with the two strains of HCV: JFH-1 replicated well in Huh-7.5.1 cells compared to HCV-RMTtri, but the opposite was seen in HuH7-K4 cells. These tendencies were observed repeatedly (Figure S1). Different replication abilities were also observed among derivatives of the HCV-RMT strain and corresponded to the colony-forming ability of the replicon constructs (Figure 2C). Immunostaining of HCV core proteins revealed that many cells (19.2%) were stained in HCV-RMTtri RNA electroporated cells compared to small number cells (0.98%) were stained in HCV-RMT with E1202G mutated RNA electroporated cells (Figure 2D).

### The supernatant of HCV-RMTtri-replicating cells was infectious to naïve HuH7-K4 cells

**Figure 3:**
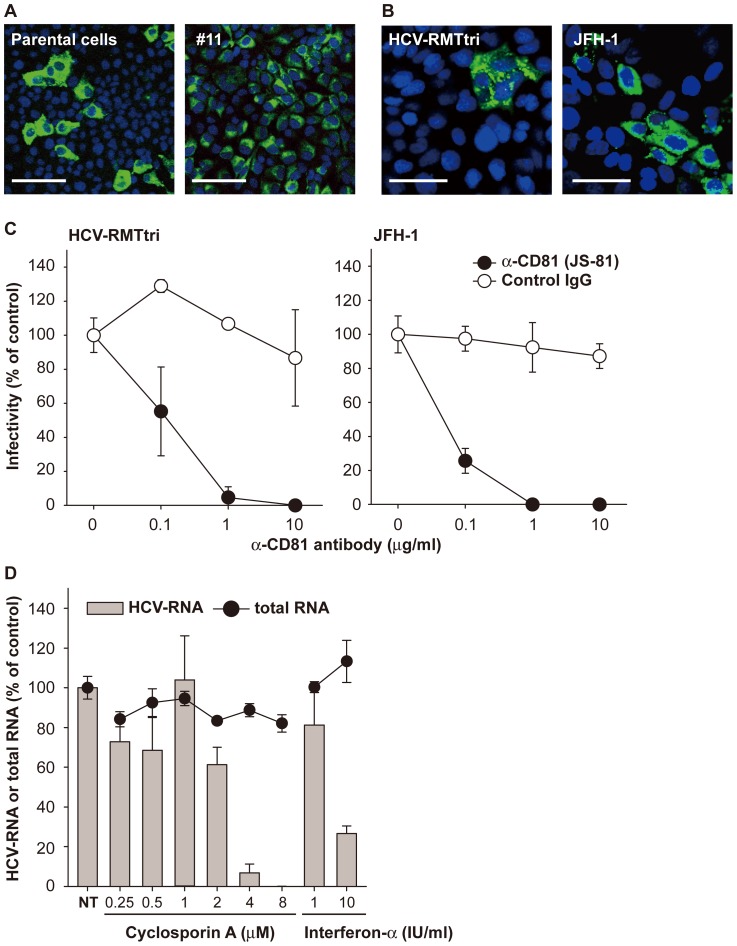
Establishment of HCV-RMTtri highly replicating #11 cell and infectivity of its supernatant on naïve HuH7-K4 cells. (A) Immunostaining for the HCV core protein in HCV-RMTtri-electroporated parental cells and the cell clone (#11) obtained by limiting dilution cloning. Scale bar  =  100 µm. (B) Immunostaining for the HCV core protein in naïve HuH7-K4 cells infected with supernatants of HCV-RMTtri- or JFH-1-replicating cells. Scale bar  =  50 µm. (C) Infection with the HCV-RMTtri supernatant was inhibited with anti-CD81 antibody in a similar manner as JFH-1. Control IgG (normal mouse IgG_1_): open circles, anti-CD81 mAb (JS-81): closed circles. Data are indicated as the mean ± S.D. (D) Replication of HCV-RMTtri in HuH7-K4 cells was inhibited by HCV replication inhibitors such as cyclosporin A and interferon-α. Drugs were added to #11 cells in 96-well plates 1 day after passaging, and cells were harvested after 72 h of treatment. NT: no treatment.

To assess the infectivity of HCV-RMTtri, we used the limiting dilution method to establish clone number 11 (#11) cells in which HCV-RMTtri was highly replicating. The percent of cells expressing the HCV core protein in #11 cells was 75.3±5.0% as seen with immunostaining, whereas the percent of parental cells expressing the HCV core protein was 6.3±2.2% (Figure 3A; the value was calculated as an average of ten observed areas). The cells maintained 1×10^8^ copies/μg total RNA of the HCV-RMTtri RNA genome. We collected the supernatants from #11 cells 2 months after cloning and HuH7-K4 cells carrying JFH-1 2 months after establishment. To evaluate infectivity, we added these supernatants to the medium of naïve HuH7-K4 cells. Cells were stained with anti-HCV core protein antibody 3 days later, and we observed core protein-positive cell foci per 0.78 cm^2^ in at least triplicate wells (Figure 3B). The calculated ffu of the supernatant was 160 ffu/ml, which was similar to that of H77 with artificially introduced adaptive mutations. This infection was inhibited by anti-CD81 antibody in a similar concentration-dependent manner as *in vitro* infection of JFH-1 (Figure 3C). #11 cells were also useful for evaluating anti-HCV agents such as cyclosporin A and interferon-α (Figure 3D) when 5,000 cells/well (96 well plate) of #11 cells were treated with inhibitors for 72 h beginning 1 day after passaging.

### *In vivo* replication abilities of HCV-RMT derivatives were inversely proportional to their *in vitro* abilities

**Figure 4:**
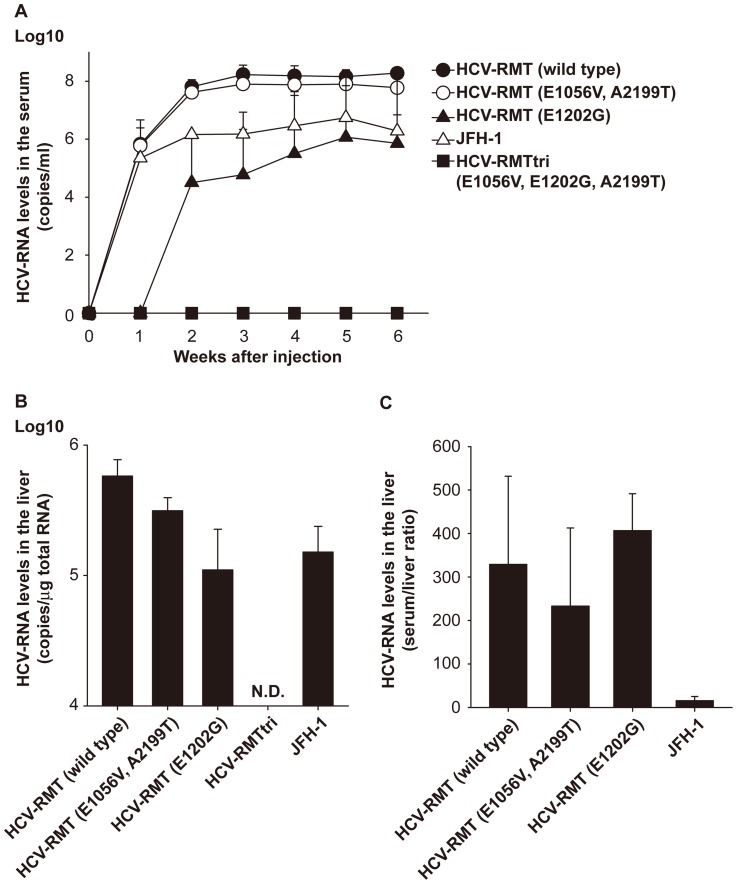
*In vivo* replication ability of HCV-RMT derivatives. (A) Change in HCV copy numbers in the serum of chimeric mice in which the HCV genome was directly injected into the livers. HCV-RMT (wild type): closed circles, HCV-RMT (E1056V and A2199T): open circles, HCV-RMT (E1202G): closed triangles, HCV-RMTtri: closed squares, JFH-1: open triangles. Data are indicated as the mean ± S.D. (B) HCV copy number in the livers. N.D.: not detected. (C) Serum/liver ratio of HCV copy number.

We assessed the *in vivo* replication abilities of HCV-RMT derivatives carrying combinations of the three adaptive mutations using chimeric mice with humanized liver. *In vitro*-generated HCV genomic RNAs were injected directly into the livers of the chimeric mice during abdominal surgery. Mice were monitored for amounts of genomic RNA in the blood once a week for 6 weeks, and virus titers in the livers were quantified after sacrifice of the mice. As shown in Figure 4A, in contrast to the vigorous *in vitro* replication ability, the clone that was most active *in vitro*, HCV-RMTtri, showed no evidence of replication *in vivo*, whereas the wild type showed replication that was comparable to the parental virus in the patient’s serum, HCG9. In addition, the double mutant (E1056V, A2199T), which showed little replication *in vitro*, showed a similar replication ability as the wild-type clone. The most positively influential adaptive mutation (E1202G) seemed to hamper its *in vivo* replication ability. Quantification of HCV genomic RNA in liver (Figure 4B, C) showed a conserved serum/liver ratio among HCV-RMT derivatives. Thus, the blood titers directly reflected the titers in liver, although the ratio was considerably different than that of JFH-1. Table 1 shows the replication abilities of derivatives and JFH-1 both *in vitro* (HuH7-K4 cells) and *in vivo* (chimeric mice), clearly showing the inversely proportional relationship between them, including the replication ability of JFH-1, which corresponds to data in a previous report [29].

**Table 1:** Relationship between the *in vitro* and *in vivo* replication ability of HCV-RMT derivatives.

Clones	*in vitro*	*in vivo*
HCV-RMT (wild type)	-	+++
HCV-RMT (E1056V, A2199T)	-	+++
HCV-RMT (E1202G)	+	+
HCV-RMTtri	+++	–
JFH-1	++	+

*in vitro* column, +++: maximum replication ability, ++: approximately 1 log lower than the maximum, +: approximately 2 logs lower than the maximum, –: no difference compared to the wild-type strain. For the *in vivo* column, +++: maximum replication ability, ++: approximately 1 log lower than the maximum, +: approximately 2 logs lower than the maximum, -: no replication. For the

## Discussion

In this report, we investigated many types of HCV replication systems using our newly cloned HCV-RMT.

The first type is replication in cultured cells as an authentic replicon construction. This system only depends on the ability to replicate in cells. HCV-RMT was the first genotype 1a clone that could be established in authentic replicon cells without artificially introduced adaptive mutations that are required by H77 [30], [31], although the three spontaneously occurring mutations (E1056V, E1202G, and A2199T) are not novel [11], [31]. Among the mutants with single mutations or a combination of these three adaptive mutations, the amounts of HCV genome and viral proteins did not reflect the colony-forming abilities (Figure 1D, E). The A2199T mutation, which least affects the colony-forming ability (no stable replicon cell line was established with this single mutation). However, combination of mutations including A2199T, triple (E1056V, E1202G and A2199T) and double (E1056V and A2199T), allowed HCV subgenomic replicon cells to produce high amounts of HCV proteins. This observation illustrates the complex nature of HCV subgenomic replicon-establishing factors, especially NS5A-related factors [32], [33]. This hypothesis requires further investigation.

The second type is replication of the virus itself in cultured cells. This system also only depends on the ability to replicate in cells as long as the presence of structural protein regions does not cause any differences. Electroporation of HCV genomes resulted in constant replication when the combination of active derivatives of HCV-RMT and HuH7-K4 cells was used; replication lasted for more than 2 months (data not shown). The order of the replication ability of mutants in cultured cells as a virus appeared to be nearly consistent with the colony-forming ability of replicons of each sequence, although some constructs with “weak” adaptive mutation(s) showed no difference from the wild type (Figure 2C). Thus, these two types of replication may be basically the same despite the different constructs. HCV-RMT derivatives replicated better than JFH-1 in HuH7-K4 cells. In contrast, replication was much less efficient in Huh-7.5.1 cells (Figure 2B), which are well known to support replication of JFH-1 [34]. These materials appear to be good tools for investigating the mechanism of HCV replication in cultured cells.

The third type is *in vitro* infection using established HCV-infected cultured cells as the source of inoculum. Because this system has more steps than the first two, the outcome is more difficult to understand. Infection systems using strains other than JFH-1 seem to be rare because the magnitude of their replication is somehow compromised [23], , in contrast to several studies examining the JFH-1 strain or its chimeric constructs with other genotypes [17]–[22]. For observation of the infection process, selection of efficiently replicating cell clones from HCV-RMT RNA-electroporated cells was required. That clone, designated HuH7-K4-#11 cells, had approximately 1×10^8^ copies/μg total RNA of the HCV-RMT genome, and more than 80% of cells were core protein positive (Figure 3A). We were able to infect naïve HuH7-K4 cells with its supernatant (Figure 3), and the infectivity reached approximately 160 ffu/ml, which was comparable to that of the artificially mutated H77 strain. Thus, our HCV-RMT strain was unique among all genotype 1a clones. We could only detect infectivity using HCV-RMTtri, likely because the abundance of HCV-positive cells was high compared to other mutants (data not shown).

Many reports investigating the cellular and/or viral factors required for *in vitro* infection of HCV have been published [36]–[45]. Almost all of these reports used a combination of JFH-1 and Huh-7.5 or Huh-7.5.1 cells. Our system using genotype 1a HCV-RMT and HuH7-K4 cells could complement these studies, considering the fact that the replication abilities of HCV-RMT and JFH-1 were quite different in the two derivatives of HuH-7 cells (Figure 2).

Among the host factors reported to influence the virus life cycle *in vitro* and *in vivo*, CD81 was the first reported receptor to be involved in the *in vitro* infection process [22], [36]-[39], and CD81 was also necessary in our HCV-RMT infection system because infection was blocked by anti-CD81 mAb (Figure 3).

Pietschmann et al. reported that the production of virus particles is impaired by replication-enhancing mutations [23]. Because we could not detect any infectivity in any supernatants from HCV-RMT derivative-electroporated cells at early times, we could not determine whether this idea applies to these derivatives. Our observation of *in vitro* infectivity only with HCV-RMTtri may be due to the balance of replication ability and budding ability. Our system may be sufficiently efficient to quantify the infectivity titer.

Other cellular factors such as lipid droplets that interact with core proteins [40]–[42] and apolipoproteins [43]–[45] have been reported previously, although their contribution to our new infection system requires further studies.

The last type of HCV replication system we investigated was *in vivo* infection. The chimpanzee model was the first animal model established for HCV infection and was frequently used in important studies despite its high cost and ethical problems. Studies using chimpanzees have revealed that *in vitro*-adapted HCV mutants require back mutation(s) at specific site(s) for efficient replication *in vivo* [46]. In our studies using chimeric mice with humanized liver, we also did not observe amplification of HCV-RMTtri, which was the most active *in vitro* mutant. In addition, we observed a clear inversely proportional relationship between the *in vitro* and *in vivo* replication abilities of each mutant (Table 1), suggesting that the same factor(s) may work in both *in vitro* replication enhancement and *in vivo* replication inhibition. Although we have not confirmed whether back mutations or other new complimentary mutations were present, the characteristics of these three mutations were clarified by analysis of these four types of replication: replicon, virus, *in vitro* infection, and *in vivo* infection. E1202G, one of the two NS3-adaptive mutations, was the most important mutation for *in vitro* replication, but it also severely hampered *in vivo* amplification. This mutation appears to impact the colony-forming ability comparable to the triple mutation, although virus replication was relatively lower than with the triple mutation. E1056V, another NS3 mutation, had a mild impact on the colony-forming ability, but it did not hamper the efficient replication of wild-type RMT *in vivo* in combination with A2199T, which seemed to have little influence on HCV replication alone except for increasing the amounts of virus genome and viral proteins in replicon cells. These two “weak” adaptive mutations provide the E1202G single mutant the ability to efficiently replicate *in vitro*. These effects may be dependent on the colony-forming ability of E1056V, the genome- and protein-increasing ability of A2199T, or both. At the same time, the weak *in vivo* replication ability of the E1202G single mutant, the replication of which was detected in only two of three mice injected and which showed a relatively low titer, was destroyed by addition of these two mutations, although the combination of these mutants had little effect on the replication ability of the wild type. These results suggest that the putative mechanism that renders *in vitro*-active clones deficient *in vivo* is not caused by a single factor such as the phosphorylation status of the NS5A protein, but a balance of many factors controlling mechanisms that are directly related to HCV replication both *in vitro* and *in vivo*.

We evaluated the amounts of HCV genome both in blood and liver, and the blood:liver ratios of replicable mutants varied little. This observation seems to be inconsistent with the hypothesis that the *in vivo* ability of *in vitro* active mutants is compromised because of adaptive mutations that make the HCV genome more replicable but impair its virion-producing ability. Whether this occurs in certain conditions or is universal must be elucidated.

Recently, Li et al. reported the efficient replication and infection of Huh-7.5 cells of a genotype 1a clone named TN with artificially introduced adaptive mutations [24]. Similar reports have been published regarding an infectious genotype 1 HCV genome in Huh-7.5 cells or their derivatives by introducing adaptive mutations or using replication enhancing reagent [23], [25]. Although these appear to be more infectious than our HCV-RMT strain, we believe that our system is valuable because of the cells we used. HuH7-K4 cells are not a derivative of Huh-7.5 cells and are apparently distinct from them in terms of the ability to support HCV replication (Figure 2).

Our newly cloned HCV-RMT strain is unique because of its vigorous replication ability in chimeric mice, compared to the first HCV strain, H77, or the JFH-1 strain that is well known for its efficient replication *in vitro*. We believe that the different levels of *in vitro* replication abilities of these HCV-RMT mutants with an inversely proportional relationship to *in vivo* replication are valuable tools for investigating the factors required for HCV replication both *in vitro* and *in vivo*.

## Supporting Information

Figure S1 *In vitro* replication ability of HCV-RMT derivative genomes. Electroporation of the generated HCV-RNA genomes of wild-type HCV-RMT (closed triangles) and HCV-RMT with triple mutations (HCV-RMTtri; closed circles) into HuH7-K4 cells. The experiments were carried out in duplicate.
(TIF)
