# Supplementary figures and images for: Isolation and Characterization of Highly Replicable Hepatitis C Virus Genotype 1a Strain HCV-RMT
Source: PLoS One. 2013 Dec 16;8(12):e82527. doi: 10.1371/journal.pone.0082527 (PMC3865021; doi:10.1371/journal.pone.0082527)

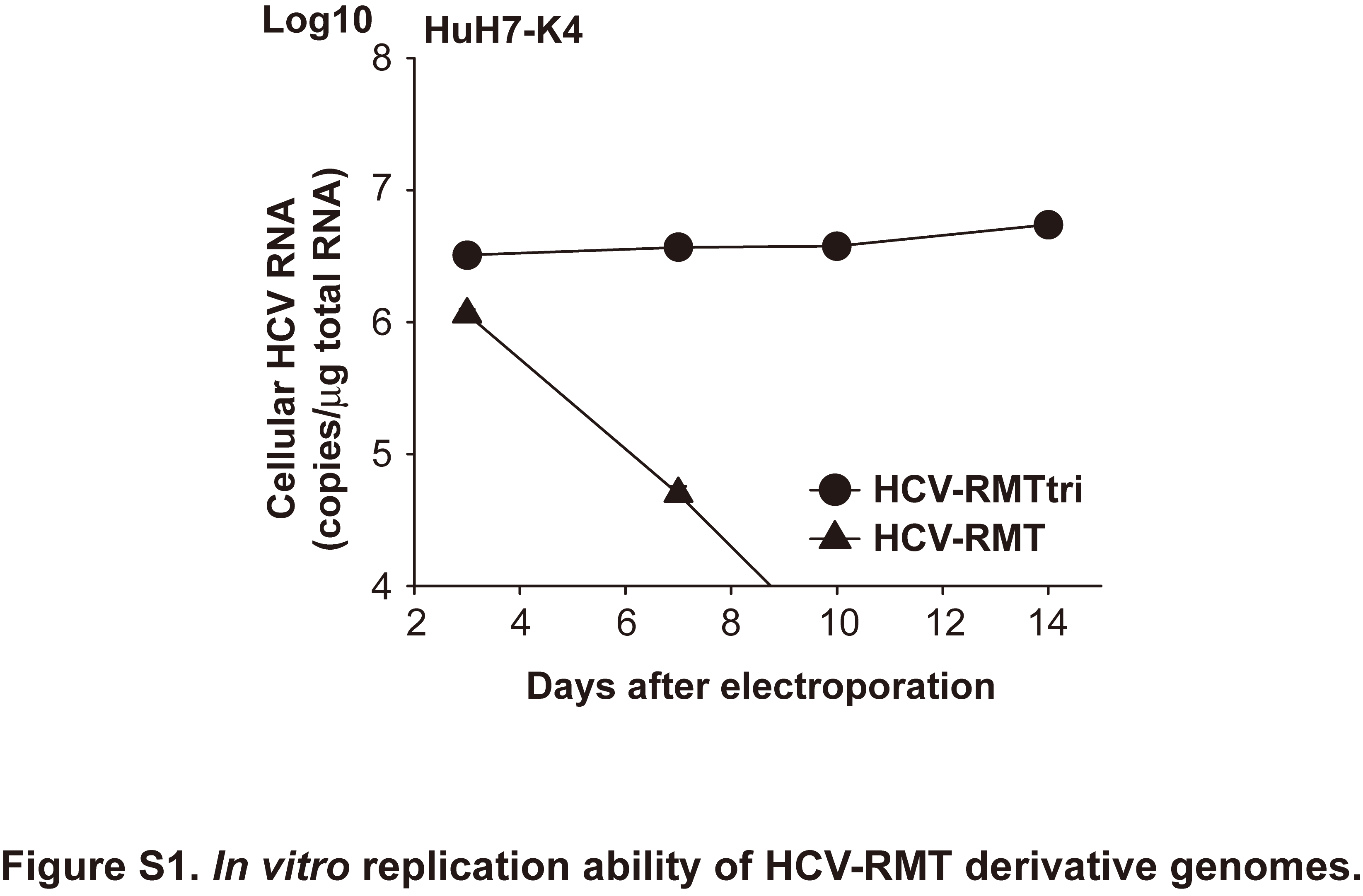

Supplement: Figure S1 — In vitro replication ability of HCV-RMT derivative genomes. Electroporation of the generated HCV-RNA genomes of wild-type HCV-RMT (closed triangles) and HCV-RMT with triple mutations (HCV-RMTtri; closed circles) into HuH7-K4 cells. The experiments were carried out in duplicate. (TIF) [file pone.0082527.s001.tif]
